# Spontaneous Bacterial Peritonitis Complicated by Loculations Requiring Alteplase

**DOI:** 10.7759/cureus.71456

**Published:** 2024-10-14

**Authors:** Tomas Lucioni

**Affiliations:** 1 Internal Medicine, Oregon Health & Science University (OHSU), Portland, USA

**Keywords:** alteplase (tpa), decompensated cirrhosis, loculated ascites, paracentesis, spontaneous bacterial peritonitis (sbp)

## Abstract

The state of healthcare is consistently being driven towards evidence-based practices to establish both optimal and consistent healthcare for all patients. From this evidence, treatment guidelines arise to meet these goals. As new data, diseases, and treatments develop, these guidelines that influence evidence-based practice are refined and redefined. However, while we have come far to understand the human body, there remain gaps in our knowledge and in the guidelines we rely on to treat our patients. We present a case of a patient with a common presentation but a rare finding where the guidelines have not yet been defined due to a lack of data. We describe a case of spontaneous bacterial peritonitis complicated by loculated ascites leading to hospital readmission. Of the patient’s two loculated collections, only one of the two nearly resolved with clearance of loculations. That collection was treated with drain placement and alteplase dwells. There has been no analysis into whether this potential treatment may improve outcomes, but there are limited cases suggesting its possible benefit.

## Introduction

The treatment guidelines for spontaneous bacterial peritonitis (SBP) are well-defined and backed by a litany of evidence. Briefly, its treatment consists of a third-generation cephalosporin (typically cefotaxime or ceftriaxone), a weight-based albumin infusion, particularly in patients with renal dysfunction, and cautious use of beta blockers [1]. Treatment of loculated ascites, however, proves to be challenging with the few case reports that exist demonstrating inadequate treatment response to the standard SBP guideline. Currently, there are no evidence-based treatment guidelines for loculated ascites, specifically regardless of cause. The majority of loculation cases reported arise from malignancy with peritoneal metastasis. In SBP, loculations typically do not form unless treated late or missed entirely, which commonly results in death [2]. The incidence of malignant ascites is about 10% [3], whereas the SBP incidence is 10-25% [4]. In hospitals, mortality associated with SBP varies but generally resides between 20-30% at diagnosis. This is accompanied by a poor overall prognosis with an estimated three-year mortality of 66.5% [5,6]. Speculatively, SBP complicated by loculations likely carries a mortality rate either on the higher end of this spectrum or beyond it, necessitating further study to establish a defined treatment pathway. Below we present a case of such a presentation and the possible role of fibrinolytics in its treatment. 

## Case presentation

A 68-year-old male with a history of decompensated cirrhosis secondary to hepatitis C (on treatment at the time of presentation), treated diffuse large B-cell lymphoma in 2019, heart failure with reduced ejection fraction (30-35%), atrial fibrillation, and chronic kidney disease stage III presented to the hospital in May of 2024 with complaints of increasing abdominal pain and chills. He had been recently discharged one month prior for heart failure exacerbation/decompensated cirrhosis and had undergone a paracentesis during that admission. CT scan at the initial presentation showed no acute findings. Diagnostic paracentesis was consistent with SBP, showing a WBC of 6856 (Table 1). He was initiated on treatment with ceftriaxone as well as albumin infusion, octreotide, and midodrine for hepatorenal syndrome, which complicated his course. Cultures ultimately grew sensitive Streptococcus dysgalactiae. Due to persistent abdominal pain and low-grade fevers, repeat diagnostic paracentesis was undertaken to ensure response to treatment, which showed an improved WBC of 659 (Table 1, 2). However, bedside ultrasound demonstrated newly appreciated multiloculated ascites. His case was discussed with infectious disease and hepatology at that time, who determined that based on his cultures and repeat paracentesis, the treatment he had received was adequate. Atypical cells were seen on two out of four of his paracenteses during his initial three-week admission, but cytology was negative on the three instances it was checked including in the atypical samples (resulting in mature lymphocytes). Ultimately, he was discharged on prophylactic ciprofloxacin.

**Table 1:** Dated list of the patient’s infectious paracentesis results, including a baseline study one month prior to his admission (4/01/2024) as well as during his subsequent admissions to the hospital.

Paracentesis Date	Location of Paracentesis	WBC	Neutrophils (%)	RBC	Culture Result	Cytology Result
4/01/2024 (Baseline)	Left lower quadrant	42	2	<3000	Negative	Not collected
5/08/2024	Not specified	6,856	90	3000	Streptococcus	Not collected
5/13/2024	Left lower quadrant	659	19	<3000	Negative	Negative
5/16/2024	Right lower quadrant	858	42	25,000	Negative	Negative
5/20/2024	Right lower quadrant	888	4	11,000	Negative	Negative
6/08/2024	Suprapubic	2,489	40	38,000	Negative	Negative
6/11/2024	Right upper quadrant	4,142	95	22,000	Negative	Negative

**Table 2:** The remaining set of the ascitic fluid parameters collected. NC signifies that the study was not collected for that procedure date. Notably, due to the different providers performing the procedures and split presentation, there are variations in what was collected. Furthermore, a complete set of results to evaluate Runyon's criteria was not checked until later in the patient’s course (5/16/2024), when he had already been on treatment. His results from 5/16, 6/8, and 6/11 were all positive by the criteria (LDH upper limit of normal 190 by our lab), but no perforation or malignancy was detected during his workup to suggest a secondary SBP. LDH: lactate dehydrogenase

Paracentesis Date	Albumin (g/dL)	Protein (g/dL)	LDH (U/L)	Glucose (mg/dL)	pH
4/01/2024 (Baseline)	<1.5	NC	NC	NC	NC
5/08/2024	1.8	NC	NC	NC	7.46
5/13/2024	NC	NC	NC	NC	NC
5/16/2024	2.4	4.4	203	86	NC
5/20/2024	<1.0	4.8	189	78	7.41
6/08/2024	2.0	5.0	202	70	7.34
6/11/2024	NC	NC	794	13	NC

**Figure 1:**
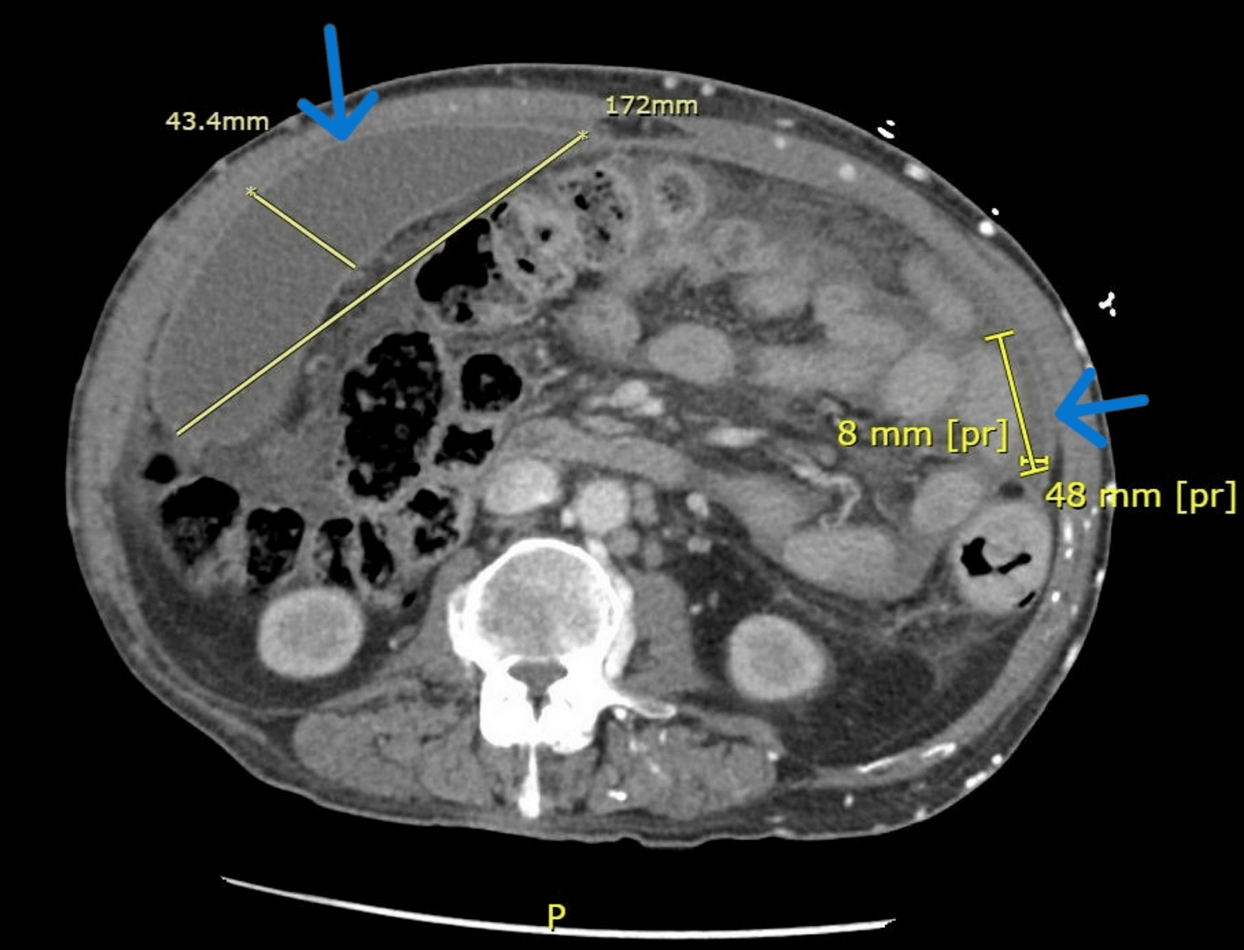
Contrasted abdominal CT scan at the patient’s readmission to the hospital. CT demonstrates a large right-sided rim-enhancing fluid collection on the left of the image. Partially imaged on the right is the most superior aspect of his smaller left-sided rim-enhancing fluid collection.

**Figure 2:**
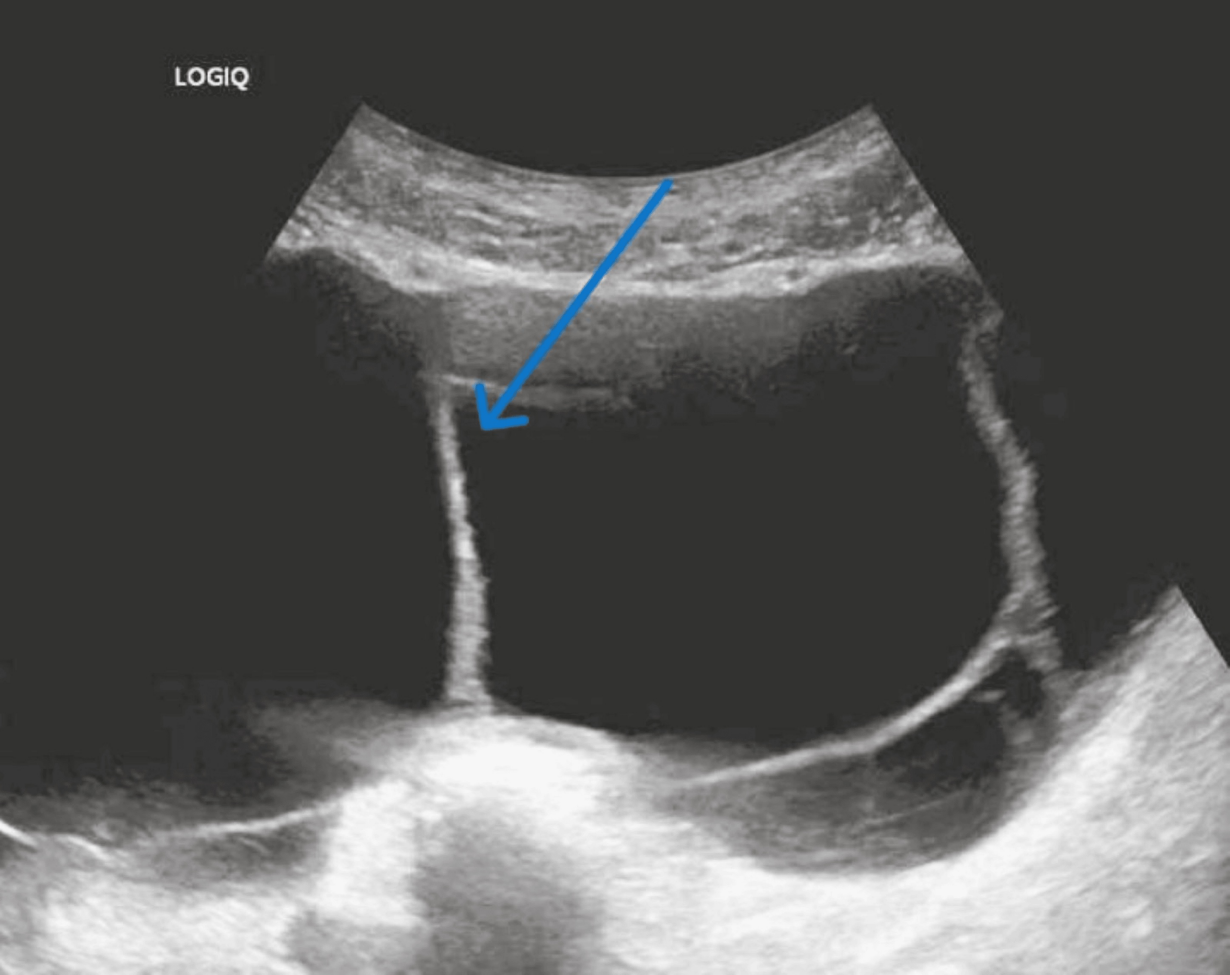
Suprapubic ultrasound demonstrating the patient’s loculations prior to drain placement and tPA dwells. Pelvic portion of his right-sided collection. tPA: alteplase

**Figure 3:**
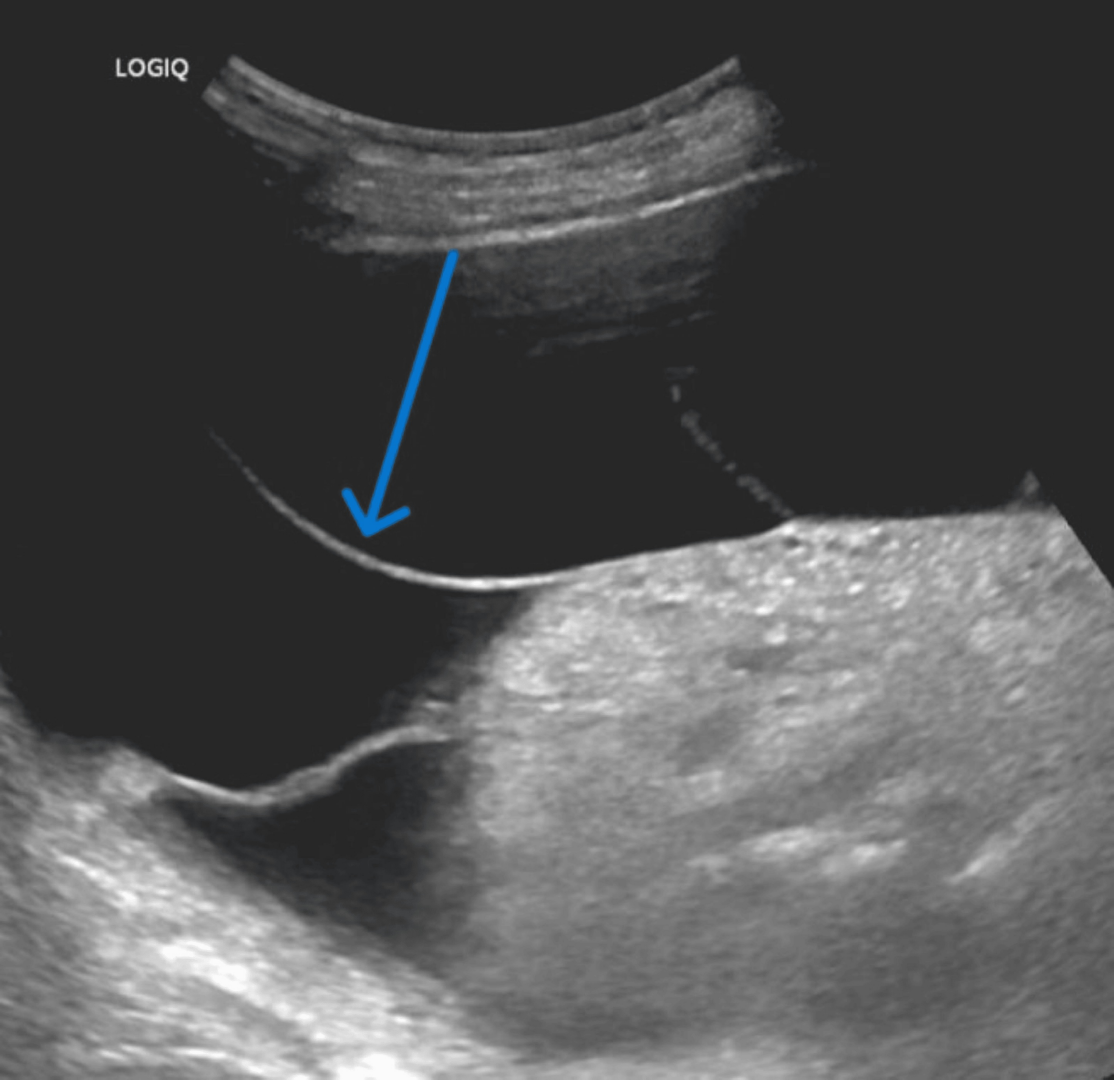
Right upper quadrant ultrasound prior to drain placement and tPA dwells. Ultrasound demonstrates a few of the patient's numerous loculations in this region with a large fluid collection. tPA: alteplase

**Figure 4:**
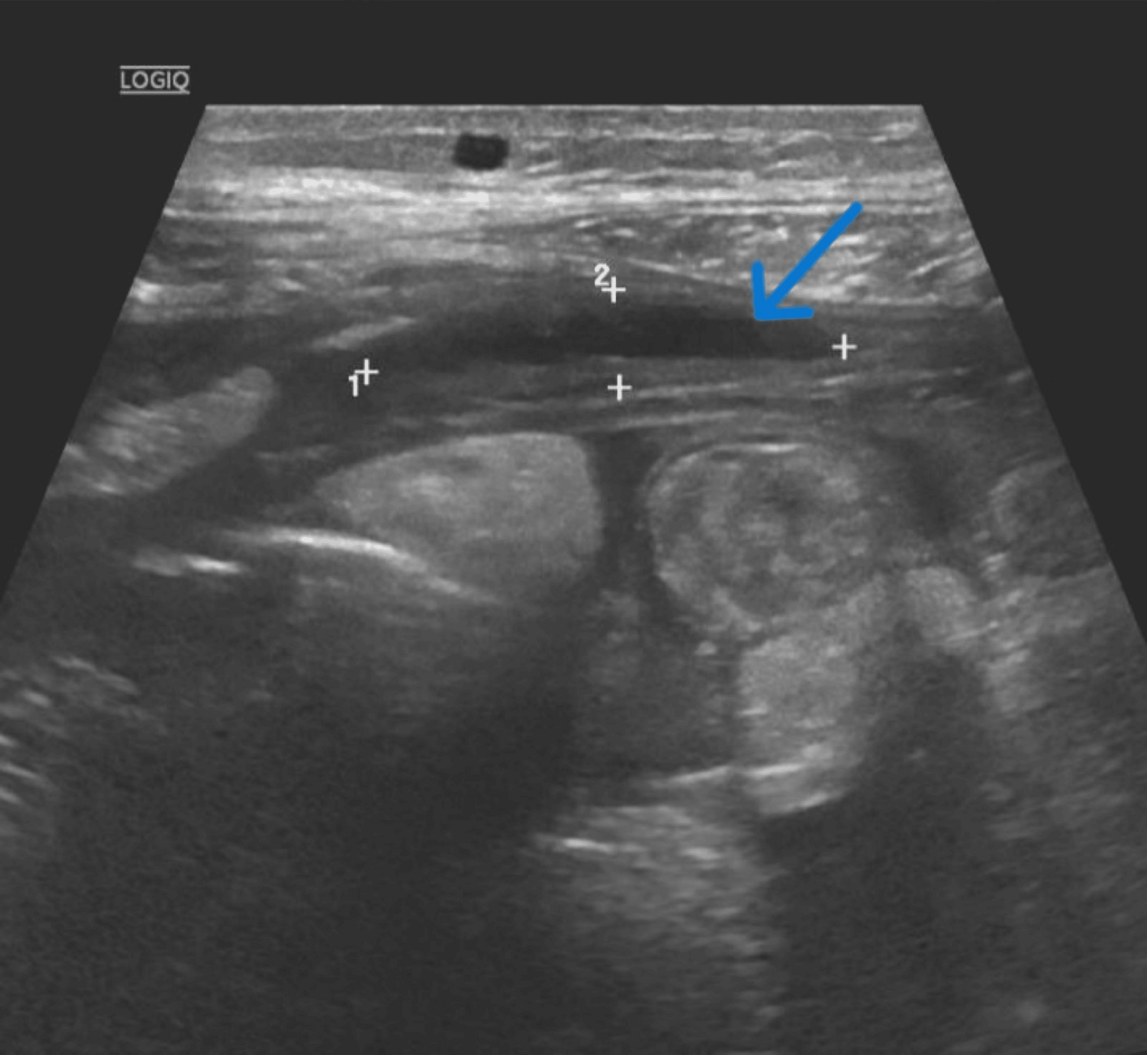
Abdominal ultrasound at the end of the patient's hospital course post-tPA dwells. Ultrasound demonstrates significantly improved fluid collection in his right abdomen without apparent loculations and partially imaged drain. tPA: alteplase

The patient returned weeks later from the clinic due to ongoing abdominal pain and concern for recurrent SBP. A CT scan at this time demonstrated increased thickening and enhancement of the peritoneum associated with moderate to large volume ascites, suggestive of peritonitis with bilateral rim-enhancing fluid collections (Figure 1). Suprapubic paracentesis showed persistent loculations (Figure 2) and WBC elevated to 2489 (Table 1, 2), so he was restarted on ceftriaxone. Cultures and cytology returned negative, and repeat paracentesis was performed after the completion of antibiotics to again ensure treatment response. Right upper quadrant paracentesis again showed significant loculations (Figure 3) and returned with a WBC further elevated to 4142 (Table 1, 2) despite appropriate antibiotics with negative culture and cytology. Infectious disease and interventional radiology (IR) were consulted. Ceftriaxone therapy was extended, and wire disruption was performed along with drain placement by IR into the right upper quadrant fluid collection (the left hemiabdomen collection was deemed too high risk for drain placement). The drain had minimal output for the 48 hours after its placement, prompting a repeat CT scan that demonstrated appropriate drain positioning with minimal change in the collection size. Due to this, alteplase (tPA) dwells were recommended, and the patient received two 4 mg dwells separated by 24 hours. Repeat ultrasound following the dwells showed resolution of the loculations and greatly improved collection in his right upper quadrant (Figure 4, initially 15.1 x 3.7 cm, decreased to 4.3 x 0.6 x 4 cm). His left hemiabdomen collection was stable in size, 7.2 x 0.8 x 3.9 cm initially, and 7.2 x 0.9 x 2.8 cm at the time of discharge. He was discharged on prophylactic cefpodoxime (chosen due to antibiotic sensitivity to recent Streptococcus dysgalactiae and readmission on prophylactic ciprofloxacin) and outpatient multispecialty follow-up. Notably, the drain was removed before discharge despite recommendations against doing so due to patient wishes.

## Discussion

While this is a single case, it demonstrates the need for a more standardized treatment approach for patients with loculated ascites. Despite appropriate antibiotic treatment and prophylaxis, he was readmitted less than one month later with either recurrent peritonitis or an incompletely treated initial infection. The potential role of fibrinolytics in the treatment of loculated ascites arose from their use in the treatment of empyema. In these cases, tPA is used in combination with dornase alfa, where it has been shown to improve fluid drainage, reduce length of stay (by six to seven days), and reduce surgical referrals [7].

The use of streptokinase has also been reported in the more commonly seen loculated malignant ascites. Lawrance et al. presented a case series where three out of four patients, with various malignancies, had loculated ascites associated with an indwelling catheter. In the three cases, 250,000 IU of streptokinase was infused over five days, leading to a resolution in two of the cases and a near resolution of loculations in the third without adverse events [8]. Delving into infectious loculations, Tripon et al. presented a case in 2020 where urokinase was used in a patient with cirrhosis secondary to hepatitis B, C, and alcohol. This patient required three drains to be placed into his loculated collections with minimal drainage. He had persistent positive ascitic cultures for E. faecium after 20 days of antibiotic treatment. At this point, the patient received 100,000 IU of urokinase in two of his drains and dwelled for 48 hours. His subsequent ultrasound showed a significant reduction in his loculations. He received one fibrinolytic treatment in total and, at five months, with assistance of low diuretic dosing, had minimal ascites and no recurrences of SBP [9].

On review at the time of this article, there exists only one other reported case where tPA was used to manage this disease process. Alkhero et al. presented a similar case to ours in 2021, also demonstrating the resolution of loculations with tPA dwells [10]. The main difference between the two cases was the dose and quantity of tPA administration (2 mg in three separate treatments rather than 4 mg in two as in our patient); however, in both cases, the loculations resolved without adverse events related to the treatment. Notably our patient also served in a way as his own control. Unfortunately, his left-sided collection was not amenable to drain placement for tPA instillation. In stark contrast to his tPA-treated collection, it showed no significant change in its size with repeat and extended antibiotic treatment. Furthermore, it showed residual and seemingly unchanged loculations on his repeat ultrasounds again in contrast to his right side.

## Conclusions

While it is much too early to make assumptions on outcome improvement, reduction in length of stay, SBP recurrence rates, or readmission rates, this case reinforces the need for further study to guide treatment guidelines. Notably, after receiving this treatment, the patient has not been re-hospitalized, shown residual signs of infection, or required subsequent paracentesis in the four months since discharge. On review of the literature, there still exists no retrospective or prospective analysis of this patient population. We implore further research that may prove that fibrinolytic agents have an important role in the management of this infectious complication.

## References

[1] Biggins SW, Angeli P, Garcia-Tsao G (2021). Diagnosis, evaluation, and management of ascites, spontaneous bacterial peritonitis and hepatorenal syndrome: 2021 practice guidance by the American Association for the Study of Liver Diseases. Hepatology.

[2] Runyon BA (2024). Diagnostic and Therapeutic Abdominal Paracentesis. UpToDate.

[3] Ilgen JS, Marr AL (2009). Cancer emergencies: the acute abdomen. Emerg Med Clin North Am.

[4] Ameer M, Foris L, Mandiga P, Mandiga P, Haseeb M (2023). Spontaneous Bacterial Peritonitis. StatPearls [Internet].

[5] Elzouki AN, Hamad A, Almasri H (2021). Predictors of short-term mortality following first episode of spontaneous bacterial peritonitis in hospitalized cirrhotic patients. Cureus.

[6] Hung TH, Tsai CC, Hsieh YH, Tsai CC (2015). The long-term mortality of spontaneous bacterial peritonitis in cirrhotic patients: a 3-year nationwide cohort study. Turk J Gastroenterol.

[7] Rahman NM, Maskell NA, West A (2011). Intrapleural use of tissue plasminogen activator and DNase in pleural infection. N Engl J Med.

[8] Lawrance N, Kibriya N, Mullan D, Laasch HU (2015). Fibrinolysis in the management of malignant ascites and nonfunctioning intraperitoneal tunneled catheters. Gastrointest Interv.

[9] Tripon S, Mayer P, Svab A (2021). Intraabdominal urokinase in the treatment of loculated infected ascites in cirrhosis. Clin Res Hepatol Gastroenterol.

[10] Alkhero M, Patel A, De Silva S (2021). Role of tissue plasminogen activator in loculated ascites. ACG Case Rep J.

